# *Mycobacterium tuberculosis* Next-Generation Whole Genome Sequencing: Opportunities and Challenges

**DOI:** 10.1155/2018/1298542

**Published:** 2018-12-09

**Authors:** Thato Iketleng, Richard Lessells, Mlungisi Thabiso Dlamini, Tuelo Mogashoa, Lucy Mupfumi, Sikhulile Moyo, Simani Gaseitsiwe, Tulio de Oliveira

**Affiliations:** ^1^College of Health Sciences, School of Laboratory Medicine and Medical Sciences, University of KwaZulu-Natal, Durban, South Africa; ^2^Botswana Harvard AIDS Institute Partnership, Gaborone, Botswana; ^3^KwaZulu-Natal Research Innovation and Sequencing Platform, University of KwaZulu-Natal, Durban, South Africa; ^4^Department of Medical Laboratory Sciences, University of Botswana, Gaborone, Botswana; ^5^Department of Immunology and Infectious Diseases, Harvard T.H. Chan School of Public Health, Boston, MA, USA

## Abstract

*Mycobacterium tuberculosis* drug resistance is a threat to global tuberculosis (TB) control. Comprehensive and timely drug susceptibility determination is critical to inform appropriate treatment of drug-resistant tuberculosis (DR-TB). Phenotypic drug susceptibility testing (DST) is the gold standard for* M. tuberculosis *drug resistance determination.* M. tuberculosis *whole genome sequencing (WGS) has the potential to be a one-stop method for both comprehensive DST and epidemiological investigations. We discuss in this review the tremendous opportunities that next-generation WGS presents in terms of understanding the molecular epidemiology of tuberculosis and mechanisms of drug resistance. The potential clinical value and public health impact in the areas of DST for patient management and tracing of transmission chains for timely public health intervention are also discussed. We present the current challenges for the implementation of WGS in low and middle-income settings. WGS analysis has already been adapted routinely in laboratories to inform patient management and public health interventions in low burden high-income settings such as the United Kingdom. We predict that the technology will be adapted similarly in high burden settings where the impact on the epidemic will be greatest.

## 1. Introduction

To curb the emergence and spread of tuberculosis (TB) drug resistance, early detection and effective treatment informed by comprehensive drug susceptibility testing (DST) are vital. It is also important to monitor and understand the development, evolution, biology, and epidemiology of TB drug resistance to inform community level or public health interventions. Molecular methods such as Xpert MTB/RIF (Cepheid, Inc. Sunnyvale, CA, USA) and the line probe assays GenoType MTBDR*plus*/*sl *(Hain lifescience, GmbH, Nehren, Germany) have considerably increased access to DST and shortened turnaround time to results. However these methods provide resistance information for a limited number of drugs.* Mycobacterium tuberculosis* WGS is an attractive method for both DST to inform treatment decisions and surveillance of drug resistance in high burden settings where capacity for routine resistance testing for everyone with TB is inadequate.

Analysis of WGS data could also be used for epidemiological investigations such as tracing of transmission chains. In this manuscript, we review how* M. tuberculosis* next-generation WGS analysis could impact prediction of drug resistance and in turn clinical management of TB especially in high burden settings. We highlight how analysis of* M. tuberculosis* whole genome sequence data could routinely provide guidance for individual treatment, tracing of transmission chains and continuous drug resistance surveillance for public health interventions. We also look at challenges or barriers to application of* M. tuberculosis *WGS analysis for routine clinical use especially in resource limited TB high burden countries.

## 2. Opportunities Presented by* M. tuberculosis* Whole Genome Sequencing

### 2.1. Investigation of Transmission Chain

The advent of next-generation sequencing (NGS) has made WGS a faster, more affordable, and increasingly accessible alternative for molecular epidemiologic studies. The data generated from WGS allows for unparalleled ability to detect genetic variation in* M. tuberculosis*. Analysis of WGS data has led to the reconstruction of* M. tuberculosis* phylogeny and this has improved our understanding of the global distribution of* M. tuberculosis* [1].

WGS has been used to answer questions about TB transmission and will, in the near future, become the routine method for* M. tuberculosis* typing because it has superior resolution to the conventional typing methodologies. Studies have shown that relatedness of* M. tuberculosis* genomes can be estimated by comparing Single Nucleotide Polymorphism (SNPs) differences between the isolates. Isolates with the smallest number of SNPs differences or shortest SNP distance would be linked, possibly representing a transmission event or cluster [2–6]. A cut-off of five SNPs or fewer for linked transmission is widely used; however a maximum of three SNPs has been suggested to represent human-to-human transmission [2–6]. Walker* et al* (2012) looked at SNP differences between epidemiologically linked pairs of isolates in the United Kingdom and none of the linked pairs exceeded five SNP difference and thus came up with the five SNP cut-off [6].

The disadvantage of this approach is that it relies on epidemiologically linked pairs. In most high burden settings especially, epidemiological links are often not available and this would make this approach problematic. Another approach has been to look at SNP differences between unlikely transmission pairs. Using this approach, a SNP difference of 0-1 has been used to define clusters based on the fact that all unlikely transmission pairs had more than two SNP differences [3]. In instances where an estimate of how long ago the transmission occurred was known, mutation rates have been used to determine a SNP cut-off [7]. This approach assumes that the mutation rate is constant over time, which is not always the case. A mutation rate of 0.003 SNPs per day has been used to determine a cut-off of ≤10 SNPs as confirmatory of transmission [7].

Analysis of whole genome data, such as clustering of SNPs, has given us an insight into the transmission dynamics as well as intra- and interpatient variations at play during outbreaks of* M. tuberculosis *[2, 3, 5, 6].

Whole genome data used in conjunction with social network analysis enabled identification of socioenvironmental factors as a driver of an outbreak in British Columbia, Canada [2]. Through detailed whole genome data analysis of another outbreak in San Francisco, cases with no obvious epidemiological connection were linked, and microevolutionary events were identified that helped to define the likely chain of transmission [8]. The resolution of WGS is superior to mycobacterial interspersed repetitive units-variable number tandem repeat (MIRU-VNTR) as evidenced by the ability of WGS to differentiate lineages of* M. tuberculosis* with identical MIRU-VNTR genotypes [2, 3, 5, 6]. WGS also allows inference about direction of transmission between the cases to be made using SNP distances, even in the absence of epidemiological data [3, 6]. This is crucial because TB outbreaks often occur in communities where epidemiological data is difficult to collect. The superior resolution of WGS has enabled us to differentiate between relapse and reinfection [9]. This is crucial for accurately evaluating treatment and prevention programmes. WGS has been used to understand transmission dynamics in a high burden setting, proof that this method could indeed have the biggest impact yet in determining* M. tuberculosis* transmission dynamics in high burden settings [7, 10].

### 2.2. Identification of Mixed Infections

Mixed* M. tuberculosis* infections are described as TB disease caused by more than one distinct* M. tuberculosis* strain. Traditionally they are identified based on at least two distinct patterns on MIRU-VNTR results.* M. tuberculosis* next-generation WGS analysis using heterozygous base calls can provide better resolution of mixed infections. However, studies have used different definitions of mixed infections based on heterozygous base calls. Presence of more than 80 and 140 heterogeneous base calls in one sample has been used to define mixed infections [7, 9]. Other studies have used classifications such as mixed base call where 38% of reads support the variant as mixed infection [3]. Mixed infections are more common in high burden settings, with 10% to 20% reported among TB patients [11, 12].

Higher rates of mixed infections have been reported among retreatment cases compared to new cases [12]. They have also been associated with poor outcomes especially where the distinct strains have different drug resistance patterns and where there is an underlying immune-suppression caused by HIV infection [13, 14]. Mixed infections may have an impact on diagnosis as evidenced by the low sensitivity (80%) of the Xpert assay for rifampicin resistance on mixed infections compared to 93% on homogenous infections [11]. Identification of mixed infections is also critical for evaluating effectiveness of tuberculosis interventions. Changes in drug resistance patterns of isolates can sometimes be explained by existence of isolates with different patterns at the same time [15]. Instances of mixed infections by strains with different DST profiles may easily be misclassified as cases of acquired resistance upon unmasking of the resistance following treatment, misinforming interventions.

### 2.3. Prediction of Drug Resistance and Understanding of Mechanisms of Drug Resistance

Unlike other molecular methods that typically target specific genes for determination of drug resistance, WGS allows for the interrogation of the entire* M. tuberculosis* genome for mutations conferring drug resistance. Mutations occurring outside the genes known to be associated with drug resistance can be identified from TB whole genomes. The likelihood of finding novel drug resistance conferring mutation is thus increased. Compensatory mutations, that is, mutations not directly involved in drug resistance but rather compensate for the fitness cost of drug resistance mutations, can be identified from whole genomes [16]. Strains with such compensatory mutations will have high fitness despite also harbouring drug resistance mutations [16]. It is through analysis of mutations in whole genomes of isolates from extensively drug-resistant tuberculosis (XDR-TB) outbreaks that we know that not all outbreaks are caused by clonal expansion of drug resistant strains, but rather some outbreaks are caused by acquired drug resistance that in many isolates appear to have been acquired independently [17, 18].

Although largely done retrospectively, WGS for determination of drug resistance has shown good concordance with conventional DST, with shorter turnaround times especially when done from early cultures [19, 20]. In one retrospective analysis, individualised drug regimens for multidrug-resistant tuberculosis (MDR-TB) and XDR-TB constructed on the basis of WGS were in close agreement with those constructed from phenotypic DST data [21]. Importantly, drug regimes constructed on the basis of WGS did not feature any drug to which phenotypic resistance was indicated, but rather WGS predicted more resistance to drugs such as ethambutol [21]. This matters because WGS would have ruled out drugs that might have been included in a treatment regimen, if decisions were based purely on information from phenotypic DST. By influencing the composition of the treatment regimen, this could lead to more effective regimens and could also reduce toxicity from unnecessary drugs.

There was strong evidence that WGS outperformed Xpert and line probe assays in terms of appropriate regimen selection [21]. Isolates with low-level resistance by WGS were susceptible by phenotypic DST probably because the critical concentrations set for DST were too high [21]. Novel or poorly defined mutations identified in phenotypically susceptible isolates were difficult to interpret [21]. This highlights the gap in knowledge of the genetic determinants of* M. tuberculosis* drug resistance that still need to be addressed. A comparison of WGS with Hain line probe assays and phenotypic DST for species identification and resistance determination for first-line drugs demonstrated comparable processing time for WGS [22]. The turnaround time for results was comparable to phenotypic DST when WGS was newly introduced into the laboratory workflow; however after successful incorporation into routine laboratory workflow, WGS results were available nine days earlier than phenotypic DST [22]. The relatively high rates of isolates with insufficient data for drug resistance determination highlight the need to further improve WGS to reduce these rates. The turnaround time for WGS results could potentially be reduced even further if efforts to apply WGS directly to clinical samples such as sputum without the need for culture are successful.

### 2.4. Whole Genome Sequencing for Continuous Drug Resistance Surveillance

In order to gauge the effectiveness of strategies to control* M. tuberculosis* drug resistance globally, accurate data on the occurrence of drug resistance is critical. WHO recommends routine DST for all TB tuberculosis patients to provide continuous surveillance of drug resistance. However most high burden countries still rely on epidemiological surveys conducted at best every five years. This is because of lack of capacity for routine DST for all TB patients; in most instances molecular methods such as the Xpert MTB/RIF are the only available methods for routine DST for suspected MDR-TB cases. Some countries have the capacity to only carry out first-line DST and have to send out samples for second-line DST to laboratories outside of the country. WGS analysis for routine drug resistance surveillance for all TB patients is an attractive avenue. However, this would have a prohibitive cost at the moment given that most of the high burden countries struggle to even afford DST for suspected drug resistant cases.

The investment in routine DST for all TB patients using WGS may not seem cost-effective in the short term. However, studies on the impact and cost effectiveness of routine WGS in high burden setting are needed to determine the feasibility of WGS in this setting. WGS analysis yields susceptibility results for both first- and second-line drugs; the data could also be an invaluable resource for understanding the epidemiology of TB in the respective countries. WGS has been used successfully to complement a drug resistance survey in Uganda where a small proportion of the isolates that were phenotypically resistant to isoniazid and rifampicin were analysed using WGS to try and understand the extent of resistance [23].

## 3. Challenges for Implementation of Whole Genome Sequencing for Routine Clinical Use

### 3.1. DNA Extraction for WGS and Quality Control

Methods that have traditionally been used for extraction of DNA from* M. tuberculosis* can in principle be employed to extract DNA for WGS. The DNA would then need to be checked for quality and concentration usually using either quantitative PCR, a spectrophotometer such as the Qubit machine, or Agarose gel electrophoresis to determine whether the quality of the extracted DNA meets the minimum standard set for the particular instrument. However, before choosing a DNA extraction method it is important to take into consideration the library preparation method or kit being used as the kit usually prescribes the minimum input DNA required for library preparation.  Table 1 summarises some of the available library preparation kits, NGS platforms with which they are compatible, and the minimum input DNA required for library preparation.

### 3.2. The Need for Culture for Whole Genome Sequencing

*M. tuberculosis* WGS has traditionally relied on growing the organism in culture. Culturing served two important purposes critical to the success of sequencing. Firstly, it ensured selective growth of* M. tuberculosis* and secondly, the growth allowed extraction of sufficient quantities of DNA for sequencing. However, this affects the turnaround time of WGS. Studies have also shown that culture methods may enrich certain strains of* M. tuberculosis* therefore affecting the population structure or clonal complexity of* M. tuberculosis* [34, 35]. The different kinds of culture media and growth conditions would therefore potentially affect the ability to detect mixed infections. This is important because, in terms of patient management, we might miss drug resistance and, in terms of molecular epidemiology, we might miss transmission events.

Recently researchers have started exploring ways to bypass the long process of growing the bacteria in culture as the starting point for WGS. Next-generation sequencing of* M. tuberculosis* from DNA extracted directly from sputum without culture, targeted amplification, or capture is challenging primarily because sputum is a complex mixture of human cells, mycobacterial cells, and oral/nasopharyngeal bacterial cells. DNA extracted directly from sputum without targeted amplification or capture will contain not only mycobacterial DNA, but also oral/nasopharyngeal bacterial DNA and large amounts of human/host DNA depending on the success of the decontamination of the sputum before the extraction. Owing to the nonspecific nature of the sequencing primers used in NGS, only a low number of reads will be from* M. tuberculosis* in cases where the concentration of* M. tuberculosis* DNA is low compared to either the host or oral bacterial DNA, as is often the case in sputum. Therefore, these approaches have been successful in sequencing* M. tuberculosis* albeit with very low coverage and depth limiting the utility of the data for downstream analysis (Table 2) [26].

To enable successful NGS with high coverage and depth, enrichment or capture methods capable of selectively capturing or enriching for* M. tuberculosis* DNA have been developed to get the DNA at concentrations and purity levels suitable for sequencing. SureSelect^XT^ Target Enrichment System (Agilent Technologies, Inc. Santa Clara, USA) is one such system (Table 2). The system has allowed high quality* M. tuberculosis* WGS without the need for culture [24, 27]. Enrichment systems could play role in ensuring that high quality WGS can be done in time to inform patient management options. However, these systems are very costly and add to the already relatively expensive WGS process. The laboratory workflows involved in these enrichment systems are also very complex and thus may remain out of reach for most programmes in regions hardest hit by TB.

A somewhat simpler, low cost approach involves depletion of the human cells or DNA using a saline wash before continuing with* M. tuberculosis* DNA extraction using ethanol precipitation (Table 2) [25]. In principle this is similar to the differential lysis protocol that has also been used successfully. The differential lysis protocol involves lysing human cells and then using DNase treatment to remove the human/host DNA followed by* M. tuberculosis* DNA extraction using a commercial kit [26]. These simpler methods tick all the boxes of an ideal extraction method; the workflow is simple and does not add much to the cost of sequencing and using the depletion of human cells approach at least, high quality sequences were generated in a clinically relevant time frame. The differential lysis protocol however has been less of a success because the proportion of reads mapping against the human genome was as high as 99% in some cases despite the attempt during DNA extraction to deplete human cells or DNA [26].

Despite the progress that has been made in developing all these methods to enable sequencing directly from sputum, we still only get good results in samples with high bacillary burden as depicted by decreasing depth and coverage with decreasing bacillary load [24]. Yet the people who could benefit most from rapid WGS-based DST are those at highest risk of morbidity and mortality, such as HIV-positive people, who are more likely to have paucibacillary disease making current methods unsuitable for such cases. Therefore much more work is needed to optimise protocols for DNA extraction and WGS directly from sputum, so that the technology can be widely applied where it is most needed.

### 3.3. Incompleteness of the Understanding of the Genetic Basis of M. tuberculosis Drug Resistance

The use of sequencing for* M. tuberculosis* drug resistance determination is limited by our current knowledge and understanding of the characterised resistance associated mutations. The current library of these characterised resistance mutations probably does not include all mutations potentially associated with the resistant phenotypes of* M. tuberculosis*. Data linking genotypic-phenotypic resistance is relatively complete for some first-line drugs such as rifampicin and isoniazid but still incomplete especially for new drugs (Table 3). In a large analysis of more than 10,000 isolates from six continents, phenotypic resistance to the first-line drugs rifampicin, isoniazid, ethambutol and pyrazinamide was predicted by WGS with sensitivities ranging from 91.3% to 97.5%, and susceptibility was predicted with specificities ranging from 93.6% to 99.0% [36]. In specific analysis of the isolates with definite phenotypic susceptibility to all four first-line drugs, WGS correctly predicted pansusceptibility in 97.9%. [36]. However, it should be noted that these estimates of predictive accuracy were based on isolates with complete phenotypic and genotypic profiles.

The susceptible phenotypes with resistance conferring mutations appeared at least based on the determined predictive performances of the mutations they harboured, to be true resistant isolates [36]. Although resistance-conferring mutations were found to be good predictors of resistant phenotypes in this study (over 90% sensitivities for first-line drugs), the mutations were linked to unexpected phenotypes in a minority of cases highlighting the geno-pheno discrepancies that still need to be resolved [36].

The phenotypically resistant yet genetically susceptible discrepancy in isolates may be explained by the fact that we are looking for mutations in genes that have already been associated with resistance, yet these phenotypes may be explained by genetic changes outside of the loci known to be associated with resistance. WGS can be used to add to the body of knowledge and characterize some of the yet to be identified and poorly characterised genetic determinants of drug resistance. To achieve this, novel mutations identified by analysis of WGS data have to be validated with phenotypic and clinical outcome data.

In an effort to contribute towards closing these gaps, a genome wide analysis of 6464 multi and extensively drug resistant* M. tuberculosis* isolates from over 30 countries identified novel resistance associated mutations including small Indels in* pncA* and large deletions in* katG [37]*. Although further functional characterisation is required to fully understand the role of these mutations in drug resistance, treatment failure, and ultimately their influence on treatment outcome, it is only through such efforts that we can one day understand the genetic determinants of* M. tuberculosis* dug resistance well enough for WGS to play a major role in TB patient management. Studies on the correlation of mutations, known and newly discovered, with treatment outcomes are urgently needed to determine the influence of the different mutations on treatment outcomes in a multidrug treatment regimen background.

### 3.4. Challenges with Handling the Large Amounts of Data from Whole Genome Sequencing

WGS produces huge amounts of data and therefore requires costly infrastructure for both storage and analysis. The analysis of WGS data also requires specialised bioinformaticians who are usually not available in clinical laboratories. However, the development of easy to use automated analysis pipelines and databases will in time allow people with minimal bioinformatic skills to analyse whole genome sequence data. Good examples of the progress that is being made in this regard are free open access online tools for rapid detection of* M. tuberculosis* drug resistance and lineage specific mutations from raw whole genome sequence data such as TB Profiler, Mykrobe Predictor TB, CASTB, KvarQ, and PhyResSE [38–40]. The tools generate results in an easy to understand format that clinicians can use for patient management [38–40]. Although still prototypes that need further testing and validation for clinical use, they are for now available for research purposes. It is a step in the right direction in terms of sorting out the analysis bottleneck that WGS data creates in clinical laboratories.

## 4. Conclusion

The future of* M. tuberculosis* WGS lies in the ability to apply the method directly to sputum, as this is the clinical material that is most commonly available. Sequencing directly from sputum samples without the need for culturing would provide a more accurate picture of the population structure of mixed infections. The relative representation of the different strains in mixed infections can be captured without the overgrowth of some strains over others due to favourable conditions of culture. This would better inform treatment and prevention interventions.* M. tuberculosis* WGS from sputum is possible and will be the way to go in the near future. This would significantly reduce the turnaround time for both resistance determinations and provide timely information about transmission dynamics.

The cost of WGS continues to go down with rapid advances to the technology. However, it remains expensive and inaccessible to high burden low-income settings, who would benefit most from the technology. We expect that in the near future* M. tuberculosis* WGS will be adapted in low-income high burden settings for periodic drug resistance surveillance to understand mechanisms of resistance and inform design of cheaper molecular diagnostics. However, if* M. tuberculosis *WGS is to really have an impact on the epidemic in high burden settings, adaptation into routine laboratory algorithms needs to occur. This adaptation will need to be preceded by upgrades in both laboratory infrastructure and key competencies such as bioinformatics, databases, and software development to provide support and allow proper handling and interpretation of the massive amounts of data generated through WGS. In conclusion,* M. tuberculosis* WGS, especially directly from sputum, will play a crucial role in the fight against the spread of TB as this significantly shortens the turnaround time to results and enables the provision of effective treatment regimen to TB patients who might be harbouring drug resistant strains.

## Figures and Tables

**Table 1:** Available library preparation kits, compatible platforms, and minimum input DNA required.

**Library Preparation kit**	**Platform/System Compatibility**	**Minimum input DNA required**
Nextera DNA Flex, Nextera XT, Nextera Mate Pair	All Illumina® platforms	1ng

Thermo Scientific MuSeek	Iron Personal Genome Machine™ and Ion Proton™ systems	100ng

Thermo Scientific MuSeek	Illumina® MiSeq and Illumina® HiSeq	50ng

Qiagen QIAseq	All Illumina® platforms	1ng

Ligation Sequencing Kit 1D, 1D^2^ Sequencing Kit,	All Oxford Nanopore Sequencing Technologies platforms	1000ng

Rapid Sequencing Kit	All Oxford Nanopore Sequencing Technologies platforms	400ng

Ion Xpress™ Plus Fragment	Ion OneTouch™ and Iron Personal Genome Machine™	100ng

**Table 2:** Summary of methods developed for DNA extraction directly from sputum samples for *M. tuberculosis* next-generation whole genome sequencing.

**Reference**	**Sample**	**Sample preparation for sequencing**	**Sequencing Platform**	**Whole genome sequencing success rate (**%**)**	%** Coverage; Average Depth of coverage**
Doyle et al., 2018 [24]	Sputum	SureSelectXT target enrichment system (Agilent technologies)	Illumina MiSeq or NextSeq sequencer	74.4%	>85%; 25X to 300X

Votinseva et al., 2017 [25]	Smear positive sputum	Saline wash, MolYsis Basic5 kit (Molzym, Germany) for depletion of human cells/DNA, Ethanol precipitation method.	Illumina MiSeq sequencer	62%	>90%; >12X

Doughty et al., 2014 [26]	Smear positive sputum	Differential lysis protocol followed by DNA extraction using the NucleoSpin Tissue-Kit (Machery-Nagel, Duren, Germany)	Illumina MiSeq sequencer	87%	20% to 99%; 0.002X to 0.7X

Brown et al., 2015 [27]	Smear positive sputum	SureSelectXT target enrichment system (Agilent technologies)	Illumina MiSeq sequencer	83%	90%; >20X

**Table 3:** Summary of the percentage phenotypic resistance that can be explained by genetic mutations at the locus of interest.

Drug	Locus	Percentage resistance accounted for by mutations	References
Rifampicin	*rpoB*	96%	[28]
Isoniazid	*katG, inhA, ahpC-oxyR*	84%	[29]
Ethambutol	*embB*	70%	[30]
Streptomycin	*rrs, rpsL*	60%-70%	[28]
Pyrazinamide	*pncA*	72%-99%	[31]
Fluoroquinolones	*gyrA*		[32]
		87% (Moxifloxacin)	
		83% (Ofloxacin)	
Aminoglycosides	*rrs*		[33]
		70%-80% (Capreomycin and Amikacin)	
		60% (Kanamycin)	
